# MoS_2_-Carbon Nanodots as a New Electrochemiluminescence Platform for Breast Cancer Biomarker Detection

**DOI:** 10.3390/bios13030348

**Published:** 2023-03-05

**Authors:** Laura Gutiérrez-Gálvez, Manuel Vázquez Sulleiro, Cristina Gutiérrez-Sánchez, Daniel García-Nieto, Mónica Luna, Emilio M. Pérez, Tania García-Mendiola, Encarnación Lorenzo

**Affiliations:** 1 Departamento de Química Analítica y Análisis Instrumental, Universidad Autónoma de Madrid, 28049 Madrid, Spain; 2 IMDEA-Nanociencia, Ciudad Universitaria de Cantoblanco, 28049 Madrid, Spain; 3 Instituto de Micro y Nanotecnología IMN-CNM, CSIC (CEI UAM+CSIC), Isaac Newton 8, Tres Cantos, 28760 Madrid, Spain; 4 Institute for Advanced Research in Chemical Sciences (IAdChem), Universidad Autónoma de Madrid, 28049 Madrid, Spain

**Keywords:** breast cancer, electrochemiluminescence, HER2, carbon nanodots (CDs), 2D molybdenum disulfide nanosheets (MoS_2_-NS), biosensor

## Abstract

In this work, we present the combination of two different types of nanomaterials, 2D molybdenum disulfide nanosheets (MoS_2_-NS) and zero-dimensional carbon nanodots (CDs), for the development of a new electrochemiluminescence (ECL) platform for the early detection and quantification of the biomarker human epidermal growth factor receptor 2 (HER2), whose overexpression is associated with breast cancer. MoS_2_-NS are used as an immobilization platform for the thiolated aptamer, which can recognize the HER2 epitope peptide with high affinity, and CDs act as coreactants of the anodic oxidation of the luminophore [Ru(bpy)_3_]^2+^. The HER2 biomarker is detected by changes in the ECL signal of the [Ru(bpy)_3_]^2+^/CD system, with a low detection limit of 1.84 fg/mL and a wide linear range. The proposed method has been successfully applied to detect the HER2 biomarker in human serum samples.

## 1. Introduction

Cancer is among the leading causes of death worldwide: in 2020, almost 10 million people lost their lives due to this disease [1]. The most common cancer among women is breast cancer [2], which represents the leading cause of death in women aged between 20 and 50 years [3]. Nevertheless, early detection that allows the premature application of an effective treatment significantly increases the probability of survival and reduces morbidity [4]. In Europe, it is estimated that 21,680 deaths per year caused by this disease could be avoided if screening coverage reached 100% [5]. However, in recent years, cancer diagnosis and treatment have been hampered by the SARS-CoV-2 pandemic [6]. Therefore, today more than ever, the importance of developing point-of-care tools for the early detection of breast cancer has been highlighted.

Breast cancer is the type of cancer that most frequently affects women today. Among the different breast cancer subtypes, there is one associated with an overexpression of the human epidermal growth factor receptor 2 (HER2) and corresponds to 20% of all breast cancer cases [7]. HER2 is a transmembrane glycoprotein, involved in the regulation of cell growth and differentiation [8], and its overexpression is related to poor cell differentiation, a short survival period, early recurrence and metastasis, strong invasion, and poor clinical prognosis [9]. This implies that it can be used as a biomarker for the diagnosis and follow-up of patients at risk of suffering from this disease or who are already under treatment. Currently, there are several methods for HER2 detection, such as fluorescent in situ hybridization (FISH), immunohistochemical (IHC) assay and enzyme-linked immunosorbent assay (ELISA) [10,11]. However, these conventional methodologies require sophisticated instrumentation, long analysis times and are laborious [12]. Consequently, the search for new technologies that can overcome these drawbacks receives a great deal of interest. In this sense, biosensor technology can represent a very attractive alternative or complementary approach to detect this biomarker because of their inherent advantages such as simplicity, low cost and possible miniaturization. In fact, recently, different types of biosensors have been developed for HER2 detection [13,14,15,16,17,18,19,20,21,22]. Considering the biorecognition element used in biosensors, antibodies are the most popular choice [15], but aptamers have emerged as an interesting alternative due to the great advantages they have over antibodies, such as higher specificity, greater design flexibility, and more stability and availability [23]. For these reasons, currently aptasensors have received increasing attention and this is an emerging area to be exploited. This technology offers the possibility of developing new simple, quick and low-cost platforms for biomarker detection as an alternative to the classic techniques above mentioned. Thus, in this work, an aptasensor for the detection and quantification of the breast cancer biomarker HER2 is presented.

Among the different transduction mechanisms in biosensors, electrochemiluminescence (ECL) is attracting scientific interest because of its high sensitivity and low background noise. ECL is a light-emitting process, whereby light emission is generated by the relaxation of excited states of luminophores that were electrochemically generated in the vicinity of the electrode surface [24]. It is an extensively used technique especially in clinical analysis, biological research and environmental applications due to its outstanding sensitivity, fast response, wide dynamic range and low background response, in addition to good stability and tunability of electrochemical methods [24,25].

This ECL emission could be reached by two main different mechanism: the ion annihilation ECL or the coreactant ECL. The most commonly used nowadays is the second one, in which the excited state of the luminophore is achieved through the reaction between the radicals generated by the luminophore and another precursor called coreactant [26]. Using the coreactant ECL, better radical ion stability and stronger ECL emission are obtained in comparison with the ion annihilation ECL, making it much more promising for the design of sensitive and reliable ECL biosensors [27].

The majority of ECL applications reported so far involve the typical coreactant ECL system consist of [Ru(bpy)_3_]^2+^ as luminophore and tripropylamine (TPrA) as coreactant [28]. However, TPrA is toxic, volatile, and less sensitive at lower concentrations [29]. For this reason, huge efforts have been made to investigate alternative coreactants for this luminophore that can be applied in biological analysis [30]. One recent discovery are carbon nanodots (CDs) [31]. These nanomaterials are a zero-dimensional fluorescent nanomaterial with interesting properties that make it a great alternative coreactant in bioanalysis, such as water solubility, high charge transfer efficiency [32,33], high stability and biocompatibility [34].

Recently, nanomaterials have been included in sensor development to improve the analytical properties of these devices. In particular, nanomaterials that can be exfoliated give well-defined sheets. Among these nanomaterials that have received increasing attention in the sensing field is molybdenum 2D disulfide nanosheets (MoS_2_-NS) due to their significantly different electrical, physicochemical, biological and mechanical properties [35]. This nanomaterial is obtained by exfoliation of bulk MoS_2_, since it has a layered structure that presents a covalent bond between Mo and S and weak interactions of van der Waals type between the adjacent sheets of the material [36]. MoS_2_-NS is clearly interesting in the development of new biosensors not only for the possibility to functionalize its surface with thiolated molecules [37,38,39,40] but also due to the possibility of developing new platforms with increased relative surface area and, therefore, with higher sensitivity. There are several biosensors reported in the literature based on the use of this nanomaterial [41,42,43,44,45,46], but it is still an emerging area that needs to be explored further. Hence, in this work, we decided to go a step forward and use MoS_2_-NS in combination with CDs for the development of a new electrochemiluminescence aptasensor for the detection of a breast cancer biomarker, HER2 protein. CDs are used as coreactants in the anodic ECL of [Ru(bpy)_3_]^2+^ and MoS_2_-NS serve as an immobilization platform for the biorecognition element, an aptamer modified with an hexalkylthiol group, which can bind MoS_2_-NS and recognize HER2 with high affinity.

## 2. Materials and Methods

### 2.1. Chemicals

HER2 DNA aptamer sequences used in this work are those reported by Liu et al. [47] and obtained by systematic evolution of ligands by exponential enrichment (SELEX). In particular, 2 synthetic thiolated aptamers (Apt-SH and Apt-TAMRA-SH) of 86 nucleotides were used. Both aptamers are modified in the 5´extreme with an hexalquilthiol (5′-SH-(CH_2_)_6_). Aptamer Apt-TAMRA-SH is also modified in its 3′ end with the fluorophore 5-carboxytetramethylrhodamine or TAMRA. Aptamers and interference DNA sequences (a non-complementary DNA sequence) used in this work are listed in Table 1 and all of them were purchased from Merk (www.merckgroup.com/es-es, Spain).

For the protocol of thiol-modified oligonucleotide reduction, a NAP-10 column of Sephadex G-25 and 1,4-dithiothreitol (DTT) were purchased from Merck (www.merckgroup.com/es-es, Spain). ELISA kit (Human ErbB2/HER2 DuoSet ELISA DY1129B) was provided by Bio-Techne R&D systems, s.l (Madrid, Spain). Sodium phosphate monobasic monohydrate (NaH_2_PO_4_·H_2_O), sodium chloride (NaCl), sodium phosphate dibasic dihydrate (Na_2_HPO_4_·2H_2_O), bovine serum albumin (BSA), human serum (from human male AB plasma, USA origin), human carcinoembryonic antigen (CEA), antigen p53, tris(2,2′-bipyridyl)dichlororuthenium(II) hexahydrate ([Ru(bpy)_3_]Cl_2_·6H_2_O) and bulk molybdenum disulfide were all provided by Merck. CDs synthesis was carried out using tiger nut milk purchased from a Spanish supermarket (Mercadona).

### 2.2. Instrumentation

For solutions preparation, purified water Millipore Milli-Q-System (18.2 MΩ·cm) was used. This equipment was supplied by Suministros Generales de Laboratorio (SGL, Barcelona, Spain).

The sterilization of all material and solutions was carried out with a Nüve OT012 autoclave before to be used.

Carbon nanodots (CDs) synthesis was performed in a CEM Discover LabMate™ microwave synthesis reactor (Matthews (NC), USA) and their purification was realized using dialysis membranes (1 kDa, Spectra/Por^®^ 6, MWCO) and 0.45 μm nylon syringe filters purchased from Suministros Generales de Laboratorio (SGL, Barcelona, Spain). For the estimation of CD concentration, CDs were freeze-dried in a freeze-dryer model Heto PowerDry LL3000 from Thermo Fisher Scientific Inc. (Waltham, MA, USA).

Carbon screen-printed electrodes (CSPEs) were used for carrying out the electrochemical and electrochemiluminescence (ECL) experiments. These electrodes integrate a carbon counter electrode, a carbon working electrode and a silver pseudo-reference electrode.

An autolab potentiostat (model PGSTAT 30), controlled with the software package GPES 4.9 or FRA 4.9, and a connector for screen-printed electrode (both from Metrohm, Madrid, Spain), were used for electrochemical measurements.

The ECL measurements were recorded in a SpectroECL controlled with the specific software DropView SPELEC version 3.2.2 E18LZ04. This instrument has a bipotentiostat/galvanostat (±4 V potential range, ±40 mA maximum measurable current). A Metrohm DropSens Si-photodiode cell (DRP-ECLPHOTODIODCELL) was used (spectral response range: 340–1100 nm). However, in the case of the spectroelectrochemiluminescence studies, a Spectrometer Cell (wavelength range: 340–850 nm) was used. The ECL equipment and both cells were provided by Metrohm (Madrid, Spain).

An airbrush equipment was used for electrode modification with MoS_2_-NS.

CDs was characterized with a JEOL JEM 2100HT transmission electron microscope from Centro Nacional de Microscopía Electrónica of Complutense University of Madrid (Madrid, Spain) operating at 200 kV. Fiji software (ImageJ 1.52n) was used for processing the obtaining images.

A FTIR (Fourier-transform infrared) Bruker IFS60v spectrophotometer and an elemental analysis equipment LECO CHNS-932 were also used for CDs characterization. Both equipments are from Servicio interdepartamental de investigación (SIdI) of Autonomous University of Madrid (Madrid, Spain).

Atomic force microscopy (AFM) images were obtained with a Nanotec Electrónica AFM system (provided by Nanotec Electrónica, Madrid, Spain), using silicon cantilevers (PPP-FM Nanosensors, 2.8 N/m nominal spring constant and a resonant frequency of 75 kHz in air), in dynamic mode and ultrapure-grade water (18.2 MΩ.cm). The acquisition and processing of images were carried out with WSxM 5.0 Develop 9.4. software [48].

For acquiring the fluorescence microscopy images, an incident light fluorescence microscope Axioskop 2 MAT (purchased from ZEISS, Oberkochen, Germany) (AxioVs40AC v. 4.1 software) with a mercury short arc lamp HBO 50 W/AC L1 (OSRAM) was used. 

A Bruker Senterra confocal Raman microscope (Bruker Optic, resolution 3–5 cm^−1^) from IMDEA Nanoscience (Spain) was used to obtain the Raman spectra using a 532 nm laser excitation, 2 mW of power and an objective 50x. Each spectrum was obtained from the average of 3 independent measurements obtained from different areas distributed throughout the sample. An Optical Olympus microscope (50X objective lens) from IMDEA Nanoscience (Madrid, Spain) was used for optical images acquiring.

SEM images have been taken with a FEI instrument (VERIOS 460), with xT microscope Control v. 5.5.2 software, provided by FEI, Netherlands.

Zeta potential measurements were carried out in a DLS Zetasizer Nano ZS (Malvern Instruments) model ZEN3600 controlled with Zetasizer Software from IMDEA Nanoscience (Madrid, Spain).

### 2.3. Procedures

#### 2.3.1. Synthesis of Carbon Nanodots (CDs)

**Scheme 1:**
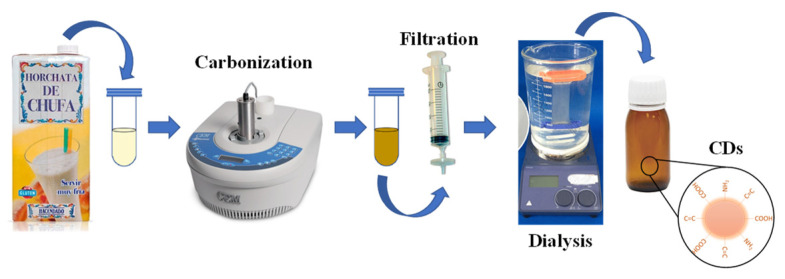
Synthesis of carbon nanodots (CDs).

CDs were prepared by the carbonization method [49,50] and following the steps of Scheme 1. Briefly, 3.00 mL of tiger nut milk was subjected to a hydrothermal treatment assisted by microwave radiation (30 min, 200 °C, 50 W and 170 psi) in a quartz flask using microwave reactor under magnetic stirring. The yellow solution obtained was allowed to cool to room temperature. After that, it was filtered with 0.45 μm nylon syringe filter and dialyzed with a Spectra/Por^®^ 6 membrane (MWCO, 1 kDa, supplied by SGL) for 1 h and 30 min to purify the CDs. Finally, the obtained CDs dissolution was stored protected from light at 4 °C. CD concentration was estimated by lyophilizing 10 mL of the purified CDs dissolution. With the weight of the solid residue, the concentration of CDs was found to be 40 mg/mL.

#### 2.3.2. Synthesis of Molybdenum Disulfide Nanosheets (MoS_2_-NS)

**Scheme 2:**
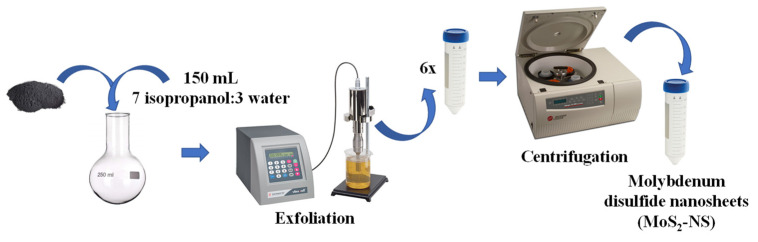
Synthesis of molybdenum disulfide nanosheets (MoS_2_-NS).

MoS_2_ nanosheets (MoS_2_-NS) were prepared following Scheme 2. A dispersion of 1 mg/mL of bulk MoS_2_ (<2 μm, 99%) in 150 mL of a 7:3 (*v*/*v*) mixture of isopropanol and water was prepared in a 250 mL round-bottom flask. Then, it was cooled down to less than 2.5 °C using a Minichiller Hubber and sonicated with an amplitude of 35% for 60 min using an ultrasonic probe (Vibracell 75115, Bioblock Scientific, 500 W, supplied by vwr) to perform the liquid-phase exfoliation step. The black suspension obtained was divided into 6 conical centrifuge tubes of 50 mL that were centrifuged for 30 min at 4000 rpm (1792× *g*) in an Allegra X-15R Beckman Coulter centrifuge (FX6100 rotor, 20 °C, purchased from Beckman Coulter Life Sciences, Pasadena, CA, USA). Finally, the olive-color supernatants were carefully collected and stored in other 50 mL conical centrifuge tubes at 4 °C. Thus, a colloidal dispersion of 0.048 mg/mL of MoS_2_ nanosheets (MoS_2_-NS) was obtained [51].

#### 2.3.3. Thiolated Aptamer, BSA and HER2 Solutions Preparation

A 0.47 µM stock solution of the thiolated aptamer (Apt-SH) was prepared following the thiol-modified oligonucleotide reduction protocol. Prior to use, the thiolated aptamer was denaturated (10 min in boiling water bath, then frozen) to achieve the correctly unfolded structure [52].

Recombinant human HER2 standard was reconstituted with 0.01 M phosphate buffer saline solution, pH 7.4, containing 137 mM NaCl, 2.7 mM KCl, 8.1 mM Na_2_HPO_4_ and 1.5 mM KH_2_PO_4_. The dilutions of this stock were prepared in 10 mM phosphate buffer (PB) 0.4 M NaCl pH 7.4 for achieved the desired concentration. All stock solutions were stored at −20 °C in aliquots of a few microliters.

A 0.1% bovine serum albumin (BSA) solution was prepared by dissolving 10 mg of BSA in 10 mL of 10 mM PB 0.4 M NaCl pH 7.4 and filtering with a 0.45 μm nylon syringe filters.

#### 2.3.4. ECL Signal of [Ru(bpy)_3_]^2+^/CD system

ECL signals on CSPE were obtained by depositing 60.0 µL of a [Ru(bpy)_3_]^2+^ solution (7 mM) and CDs (8 mg/mL) in 0.2 M PB pH 8. Then, a cyclic potential scan from 0.60 V to 1.30 V at a scan rate of 10 mV/s was applied. The produced ECL signal (amplified x10) and cyclic voltammogram were registered at the same time.

In the case of 3D electrochemiluminescence spectra of [Ru(bpy)_3_]^2+^ (7 mM) and CDs (8 mg/mL) system, the following conditions were applied: integration time: 3000 ms, scans to average: 3 and boxcar: 4. The produced ECL signal was collected simultaneously with the cyclic potential scan application.

#### 2.3.5. Biosensor Electrochemical Characterization

Electrochemical impedance spectroscopy (EIS) and cyclic voltammetry (CV) measurements were carried out for the biosensor development characterization. Cyclic voltammograms were registered from −1.0 to 1.2 V at a scan rate of 0.1 V/s. EIS data were acquired in the frequency range from 100 kHz to 0.01 Hz, with a sinusoidal voltage perturbation of 10 mV amplitude and applying a potential of 0.11 V [53]. Both measurements were registered in 10 mM Fe(CN)_6_^3−^ and 10 mM Fe(CN)_6_^4−^ as redox probe in 0.1 M PB pH 7 solution. The obtained EIS data was plotted in a Nyquist plot (-Z’’ vs. Z’) and fitted to a Randles equivalent circuit which consists of the charge transfer resistance (R_ct_), the solution resistance (R_s_), a Warburg impedance element (Z_W_) and a constant phase element (CPE) that models the non-ideal behavior of the double layer capacitance.

#### 2.3.6. HER2 Aptasensor Development

Molybdenum Disulfide Nanosheet Electrode Modification

CSPEs were modified with molybdenum disulfide nanosheets (CSPE/MoS_2_-NS) by spraying them on to the working electrode surface with an airbrush for one minute with an approximate flow of 200 µL/min. For allowing the fast evaporation of the solvent, the electrodes were heated at 90 °C on a hot plate during the process. After the nanostructuring, the electrodes were kept at room temperature overnight.

Immobilization of Thiolated Aptamer on Molybdenum Disulfide Modifed Cspes

Electrodes previously modified with molybdenum disulfide nanosheets (MoS_2_-NS) were subsequently modified with 10.0 µL of 0.47 µM of thiolated aptamer (Apt-SH) by drop casting and were kept at room temperature for 72 h (CSPE/MoS_2_-NS/Apt-SH). After that, they were washed with Milli-Q water to remove unabsorbed materials.

Blocking with BSA

CSPE/MoS_2_-NS/Apt-SH electrodes were subsequently blocked with 0.1% bovine serum albumin (BSA) in 10 mM PB 0.4 M NaCl pH 7.4 for 30 min [52]. Afterwards, they were washed with Milli-Q water to remove unabsorbed material.

Incubation with HER2 Protein

Finally, CSPE/MoS_2_-NS/Apt-SH/BSA electrodes were incubated in humid chamber (2 h at 37 °C) with 10.0 µL of the analyte protein (HER2) and washed with 10 mM PB 0.4 M NaCl pH 7.4 to remove unabsorbed material.

To obtain the calibration plot, different tested HER2 concentrations were used (428 fg/mL, 855 fg/mL, 1.71 pg/mL, 3.42 pg/mL and 13.7 pg/mL).

In the case of the interference study, CSPE/MoS_2_-NS/Apt-SH/BSA electrodes were incubated with 10.0 µL of different solutions containing HER2 (final concentration of 1.71 pg/mL) plus the different possible interferences (non-complementary DNA sequence, CEA and p53) at a final concentration of 1 µM, 100 ng/mL and 1 ng/mL for non-complementary DNA sequence, CEA and p53, respectively.

Electrochemiluminescence Detection

ECL emission was obtained by applying a cyclic potential scan from 0.50 V to 1.40 V at a scan rate of 10 mV/s in 60.0 µL of 7 mM [Ru(bpy)_3_]^2+^ and 8 mg/mL CDs in 0.2 M PB pH 8. The produced ECL signal (amplified ×10) and cyclic voltammogram were registered at the same time. Finally, the ECL aptasensor response was normalized with the blank (CSPE/MoS_2_-NS/Apt-SH/BSA) as a percentage:

$$Normalized\;ECL\;\left(\%\right)=\frac{\left(ECL\;signal-blank\;ECL\;signal\right)}{blank\;ECL\;signal}\times 100$$

HER2 Detection in Spiked Human Serum Samples

To confirm the applicability of the developed aptasensor as well as to study the matrix effect, human serum samples spiked with HER2 protein were used at a final concentration of 1.71 pg/mL HER2 using as solvent human serum. 10.0 µL of the solution were incubated on the developed platform (CSPE/MoS_2_-NS/Apt-SH/BSA) in humid chamber (2 h at 37 °C) and then, washed with 10 mM PB 0.4 M NaCl pH 7.4 to remove unabsorbed material. Then, ECL detection was perform as we described previously. Finally, the concentration of HER2 was calculated interpolating the normalized ECL signal value obtained for the spiked human serum in the calibration plot.

## 3. Results and Discussion

**Scheme 3:**
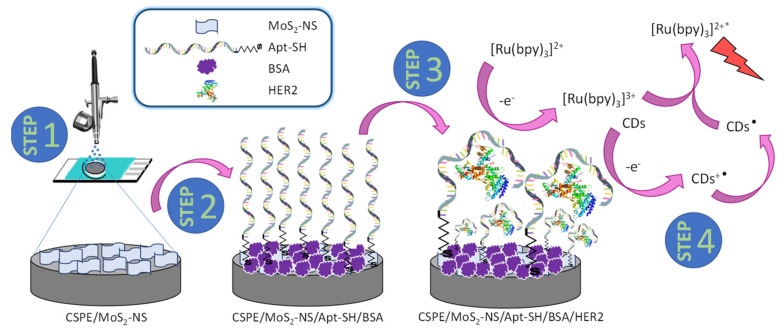
Aptasensor design.

In this work, we present a new electrochemiluminescence aptasensor for the early diagnose of breast cancer, based on the use MoS_2_-NS and CDs. Specifically, MoS_2_-NS are used as an immobilization platform for the thiolated aptamer through the thiol group, which tend to bind to MoS_2_-NS [37,38,39,40,46], and CDs are used to obtain the ECL signal, acting as coreactants in the anodic ECL of the luminophore [Ru(bpy)_3_]^2+^. In this sense, the use of [Ru(bpy)_3_]^2+^/CD system has been previously reported by us to detect DNA hybridization event on gold screen-printed electrodes-based DNA biosensor [49,50]. Hence, based on these successfully studies, in the present work we decided to go a step forward and propose to use the ECL system [Ru(bpy)_3_]^2+^/CDs to detect the highly specific protein–aptamer interaction. Through the combination of both nanomaterials, we have developed a simple, rapid aptasensor whose development steps are depicted in Scheme 3. Firstly, nanostructuration of CSPEs by spraying MoS_2_-NS was carried out (step 1). Secondly, the nanostructured surface with MoS_2_-NS is modified with the thiolated aptamer (Apt-SH) and subsequently blocked with bovine serum albumin (BSA) (step 2). Then, the modified electrode is incubated with the HER2 protein and Apt-SH recognize HER2 epitope peptide with high affinity (step 3). Finally, selective aptamer-HER2 binding is carried out by measuring the anodic ECL signal of the [Ru(bpy)_3_]^2+^ using CDs as coreactants (step 4).

### 3.1. Synthesis and Characterization of Nanomaterials: CDs and MoS_2_-NS

CDs were obtained using a green chemistry approach by treating tiger nut milk under a hydrothermal treatment in a microwave reactor [49,50] (see Scheme 1 of experimental section). Quasi-spherical nanoparticles with an average size of (7.2 ± 1.4) nm were obtained. Elemental analysis and infrared absorption spectroscopy confirm their composition and surface functional groups (see Figure S1) [49]. Zeta potential measurements gave an average value in aqueous solution of −6.7 ± 0.3 mV. This negative value of zeta potential is probably due to the surface contained carboxylate groups on the CDs.

MoS_2_-NS were prepared from bulk MoS_2_ (see Scheme 2 in Experimental section) [51]. SEM images (Figure S2A,B) show the flakes (nanosheets) of MoS_2_ with a typical lateral size ranging from 300 to 800 nm. The extinction spectrum (Figure S2C) shows the characteristic A and B excitons of MoS_2_ suspensions at 624 and 685 nm, respectively, originated by the direct band gap transition of the Brillouin zone at the K point. In addition, the Raman spectrum (Figure S2D) shows two representative bands at 380 and 406 cm^−1^ assignable to the first-order Raman active modes E^1^_2g_ and A_1g_ of MoS_2_, respectively, which are more clearly observed in the inset. The E^1^_2g_ mode is the opposite in-plane vibration of two S atoms with respect to the Mo atom, while the A_1g_ mode is associated with the out-of-plane vibration of the S atoms in opposite directions [54]. The presence of these two bands confirms that the metallic center has a prismatic trigonal coordination, being, therefore, the semiconductor polymorph 2H-MoS_2_ [36].

### 3.2. ECL Signal of [Ru(bpy)_3_]^2+^/CD system

**Figure 1:**
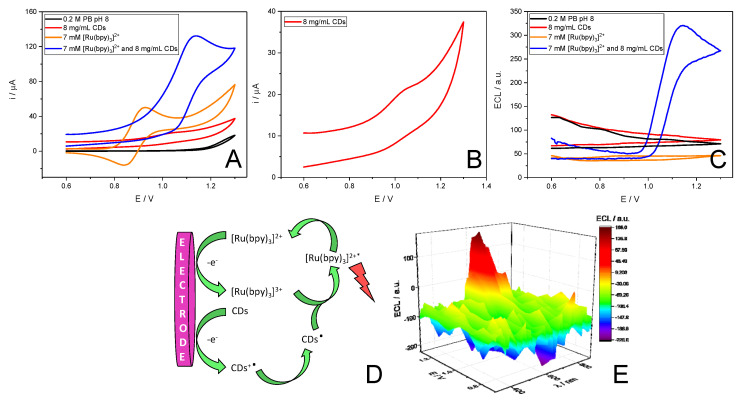
(**A**) Cyclic voltammograms and (**B**) magnification of the CD cyclic voltammogram. (**C**) ECL signals of a CSPE in a solution of: electrolyte (0.2 M PB pH 8) (black line), 8 mg/mL CDs (red line), 7 mM [Ru(bpy)_3_]^2+^ (orange line) and 7 mM [Ru(bpy)_3_]^2+^ with 8 mg/mL CDs (blue line) in 0.2 M PB pH 8. (**D**) Proposed mechanism for the [Ru(bpy)_3_]^3+^/CD system. (**E**) 3D ECL spectrum of [Ru(bpy)_3_]^2+^/CD system. These measurements were performed using 0.2 M PB pH 8 as electrolyte and applying a cyclic potential scan from 0.60 V to 1.30 V with a scan rate of 10 mV/s.

CDs can act as coreactants in the anodic ECL of the luminophore [Ru(bpy)_3_]^2+^ using gold substrates as we have previously described [49,50]. Based on this premise, we study the possibility of using this system also in CSPEs. For this purpose, we recorded the cyclic voltammograms (Figure 1A) and the resulting ECL signal (Figure 1C) of the CDs (red line), [Ru(bpy)_3_]^2+^ (orange line) and the mixture of both species (blue line) in 0.2 M PB pH 8 at CSPEs. As can be observed in Figure 1A, the cyclic voltammogram of [Ru(bpy)_3_]^2+^ presents the expected reversible redox process of [Ru(bpy)_3_]^3+^/[Ru(bpy)_3_]^2+^system while CDs present an oxidation process close to 1.0 V (see Figure 1B). When both species are used ([Ru(bpy)_3_]^2+^/CDs), the intensity of the [Ru(bpy)_3_]^2+^ oxidation peak increases concomitantly as the cathodic peak disappear which is characteristic of an electrocatalytic process. Moreover, only in the case of using the mixture of both species ([Ru(bpy)_3_]^2+^/CDs) an ECL signal can be observed. These results seem to suggest that CDs can act as coreactants in the [Ru(bpy)_3_]^2+^ anodic ECL, with a similar role of tri-n-propylamine (TPrA) in the [Ru(bpy)_3_]^2+^/TPrA ECL system. Therefore, we propose an “oxidative–reduction” coreactant ECL pathway mechanism for the [Ru(bpy)_3_]^2+^/CD system (see in Figure 1D). [49,50]. During the anodic scan, CDs and [Ru(bpy)_3_]^2+^ are both oxidized. Then, CDs can be oxidized by via chemical oxidation of the oxygen-containing units by electrogenerated [Ru(bpy)_3_]^3+^ ([Ru(bpy)_3_]^3+^ + CDs → [Ru(bpy)_3_]^2+^ + CDs^+·^) [31] or by the direct electro-oxidation of the nanomaterial (see inset of Figure 1A). The latter possibility is not represented in Figure 1C to ensure the clarity of the scheme. Immediately after, oxidized CDs (CDs^+·^) undergoes chemical decomposition to form the related reducing agent (CDs^·^) that further reduces [Ru(bpy)_3_]^3+^ generating the excited state [Ru(bpy)_3_]^2+*^, which is unstable and decays to the ground state producing an anodic red ECL emission [26,31]. To verify this proposed ECL mechanism, a 3D ECL spectrum of the [Ru(bpy)_3_]^2+^/CD system was recorded. As can be seen in Figure 1E, an ECL emission peak centered at 626 nm was observed, which is comparable to the fluorescence spectra of the [Ru(bpy)_3_]^2+^ (λ_max_ at 610 nm) (Figure S3). This result indicates that the ECL emission obtained from the [Ru(bpy)_3_]^2+^/CD system can be attributed to the electronic transition of the excited state of [Ru(bpy)_3_]^2+*^ to [Ru(bpy)_3_]^2+^ [55], suggesting that [Ru(bpy)_3_]^2+^ acts as luminophore and CDs as coreactant in this ECL system and confirms the previously proposed mechanism. The apparent 16 nm red shift of the ECL spectrum compared to the fluorescence spectra can be attributed to the different conditions in which both are registered: (1) an inner filter effect due to the high concentration of [Ru(bpy)_3_]^2+^ used for the ECL experiments (7 mM) respect to the one used for the fluorescence measurement (20 µM) [56]; (2) the different equipment (with different resolutions, slit widths, etc.) used for registering the fluorescence and ECL spectra [57].

**Figure 2:**
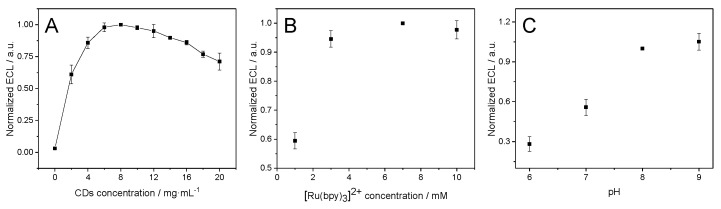
Normalized ECL signals of [Ru(bpy)_3_]^2+^/CD system obtained by varying (**A**) the concentration of CDs (from 0 to 20 mg/mL) and fixing the concentration of [Ru(bpy)_3_]^2+^ to 7.0 mM in 0.2 M PB pH 8, (**B**) the concentration of [Ru(bpy)_3_]^2+^ (1, 3, 7 and 10 mM) and fixing CD concentration to 8 mg/mL in 0.2 M PB pH 8, and (**C**) the pH value (6, 7, 8 and 9) of 0.2 M PB using a 7 mM solution of [Ru(bpy)_3_]^2+^ and 8 mg/mL of CDs. ECL signals were obtained by applying a cyclic potential scan from 0.60 V to 1.20 V or 1.30 V in CSPEs with a scan rate of 10 mV/s. ECL values are obtained from three replicate measurements.

In order to optimize the ECL signal, the influence of [Ru(bpy)_3_]^2+^ and CD concentrations in the ECL signal was studied by using a fixed concentration of [Ru(bpy)_3_]^2+^ (7.0 mM) and increasing CD concentration (Figure 2A) or a fixed concentration of CDs (8 mg/mL) and increasing [Ru(bpy)_3_]^2+^ (Figure 2B). As can be seen in Figure 2A,B, on increasing the concentration of CDs the ECL signal increases reaching a maximum at 8 mg/mL and then there is a decrease. In the case of the [Ru(bpy)3]2+, there is an increase in the ECL signal on increasing its concentration up to 7.0 mM and then level off. The excess of a coreactant as CDs may cause a decrease in the signal because it can affect the optical properties of the system. This behavior is quite similar to that observed for other coreactants [26]. These conditions were selected for the future experiments. pH influence was also studied in the ECL signal. The obtained results show an increment in the ECL signal with the pH increment value from 6 to 9 (Figure 2C). Considering that pH values higher than 9 should not be used (an increase in the ECL background signal can be observed caused by the hydroxide ions) [26], a pH value of 8 was selected for the following experiments. Therefore, pH 8 and a concentration of 7.0 mM and 8 mg/mL for [Ru(bpy)_3_]^2+^ and CDs, respectively, were considered as the best conditions for the ECL experiments.

Once we confirmed the possibility of using the ECL [Ru(bpy)_3_]^2+^/CD system and fixed the best conditions to obtain the ECL signal, the next step in this work was to study the possibility of applying this system to detect the HER2 biomarker.

### 3.3. Aptasensor Development and Characterization

As it described above, the first step in the aptasensor development is the nanostructuration with MoS_2_-NS by spraying on the carbon screen-printed electrode (CSPE) surface. Then, the optimized concentration of thiolated aptamer (Apt-SH) is immobilized on the nanostructured electrode (CSPE/MoS_2_-NS) due to the interaction of thiolated group and MoS_2_ lattice [37,38,39,40,46]. In order to prepare reproducible devices, each step followed in the development as well as the final platform was characterized by different techniques such as scanning electron microscopy (SEM), atomic force microscopy (AFM), fluorescence microscopy, cyclic voltammetry (CV) and electrochemical impedance spectroscopy (EIS).

**Figure 3:**
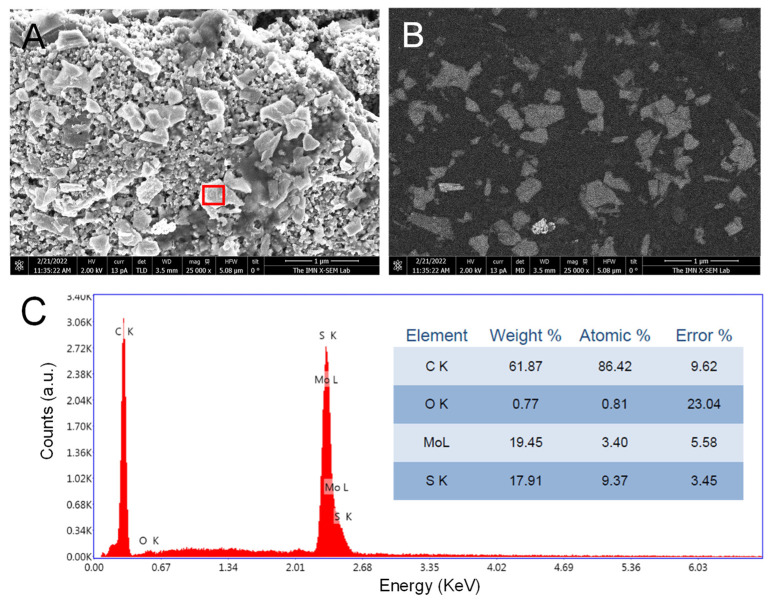
(**A**) Secondary and (**B**) backscattered electrons SEM images of CSPE/MoS_2_-NS. (**C**) EDX spectra on the MoS_2_-NS area marked on Figure 3A.

SEM images of bare CSPE (Figure S4) and CSPE/MoS_2_-NS (Figure 3A,B) were obtained. Secondary electrons images (Figure 3A and Figure S4A) are useful for the inspection of the topography of the sample´s surface while the backscattered electrons images (Figure 3B and Figure S4B) provide differences in atomic number of materials placed on and below the surface of the sample. Considering this information, the MoS_2_ nanosheets can be clearly distinguished in Figure 3B (clear gray) from the CSPE (dark gray) (Figure S4B). Moreover, EDX spectra (Figure 3C) obtained from the MoS_2_-NS area marked on Figure 3A shows the peaks corresponding to molybdenum and sulfur. Due to the absence of any element different from carbon, no contrast is observed in Figure S4B. These results point out to CSPE nanostructuration with MoS_2_-NS.

**Figure 4:**
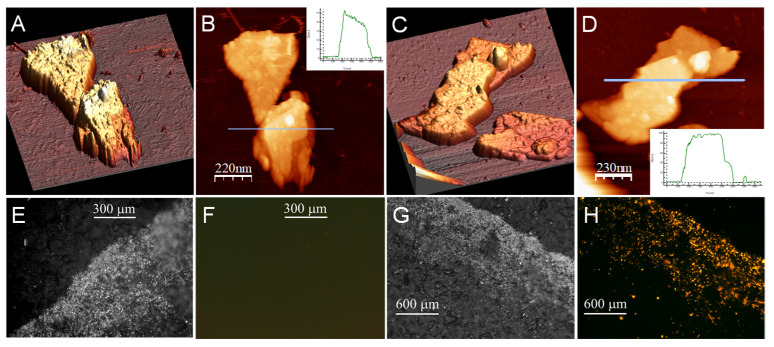
(**A**–**D**) AFM topography images of (**A**,**B**) HOPG/MoS_2_-NS and (**C**,**D**) HOPG/MoS_2_-NS/DNA. Optical (**E**,**G**) and fluorescence (**F**,**H**) microscopy images of (**E**,**F**) CSPE/MoS_2_-NS (100×) and (**G**,**H**) CSPE/MoS_2_-NS/Apt-TAMRA-SH (50×).

Figure 4A–D shows the AFM topography of several MoS_2_-NS on CSPE before (A and B) and after (C and D) the modification with the aptamer. Root mean square (RMS) roughness average of 7 MoS_2_-NS before aptamer modification is (2.07 ± 0.863) nm. This parameter increases to (3.03 ± 1.34) nm for CSPE/MoS_2_-NS/DNA. This gain in coarseness points out to the immobilization of the aptamer. Nevertheless, to demonstrate the immobilization of the aptamer upon the surface of the nanostructured electrode, fluorescence microscopy was used (Figure 4E–H). In this case, the aptamer used is modified with a fluorophore TAMRA (Apt-TAMRA-SH) (see Table 1). As can be observed in Figure 4H, only when the nanostructured electrode is modified with DNA labeled with a fluorophore (CSPE/MoS_2_-NS/Apt-TAMRA-SH) fluorescence is observed, confirming aptamer immobilization.

**Figure 5:**
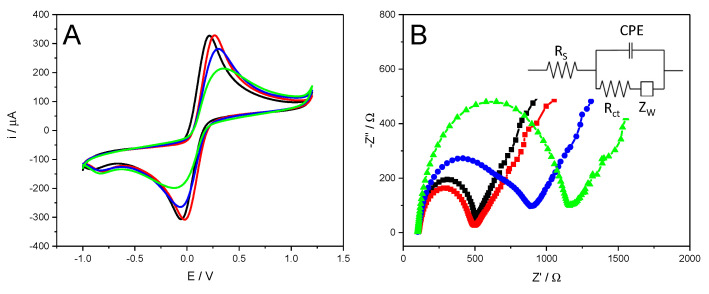
(**A**) CV and (**B**) EIS behaviors of CSPE (black curve), CSPE/MoS_2_-NS (red curve), CSPE/MoS_2_-NS/Apt-SH (blue curve) and CSPE/MoS_2_-NS/Apt-SH/BSA (green curve) in 10 mM Fe(CN)_6_^3−^ and 10 mM Fe(CN)_6_^4−^ in 0.1 M PB pH 7.

Since CV and EIS can be an effective method for probing the process of electrode modification [58], we employed them to achieve a further characterization of the biosensing platform. Figure 5A shows the obtained cyclic voltammograms of CSPE before (black curve) and after nanostructuration with MoS_2_-NS (red curve). As can be seen, the characteristic oxidation/reduction peaks of the Fe(CN)_6_^3−^/Fe(CN)_6_^4−^ redox couple is observed in both case, with a similar peak separation (ΔEp). After incubation with thiol-modify aptamer (blue curve), an increase of 339 mV in the ΔEp as well as a decrease of anodic and cathodic peak currents are noticed. These phenomena manifest a hindering of electron transfer, which is a result of the electrostatic repulsion between the negatively charged aptamer and the redox couple negatively charged [53,59,60], and suggests the aptamer immobilization on MoS_2_-NS modified electrode. After blocking with BSA (green curve), a shift of the reduction peak to a more negative potential as well as a slightly shift towards anodic potentials the oxidation peak (ΔEp = 384 mV) are observed. In addition, in both cases a significantly lower current density is observed which demonstrates the successful blocking of non-specific binding sites on the electrode surface [53,60].

To study the interface properties of each construction step of the aptasensor EIS was also registered [58]. The semi-circle diameter of a Nyquist plot equals to the value of electron-transfer resistance (R_et_) which controls the electron-transfer kinetics of the redox probe at the electrode interface [58]. The Nyquist plot (Figure 5B) for the bare CSPE (black curve) shows a charge transfer resistance of 399 Ω and in the case of the CSPE/MoS_2_-NS (red curve) a value of 388 Ω pointing out an improvement in the charge transfer after nanostructuring the electrode. After the aptamer modification, the semicircle diameter increases (785 Ω), caused by the electrostatic repulsion between the negatively charged aptamer layer and the negatively charged Fe(CN)_6_^3−^/Fe(CN)_6_^4−^ redox couple [53,59,60], thus confirming the aptamer immobilization on the CSPE/MoS_2_-NS. Finally, a further increase of charge transfer resistance to 1062 Ω was observed after blocking the surface with BSA, as the presence of BSA on the electrode surface blocks nonspecific binding and hinders the interfacial electron transfer [53,60]. EIS results were consistent with the observations through CV, which confirm the successful development of the aptasensor.

**Figure 6:**
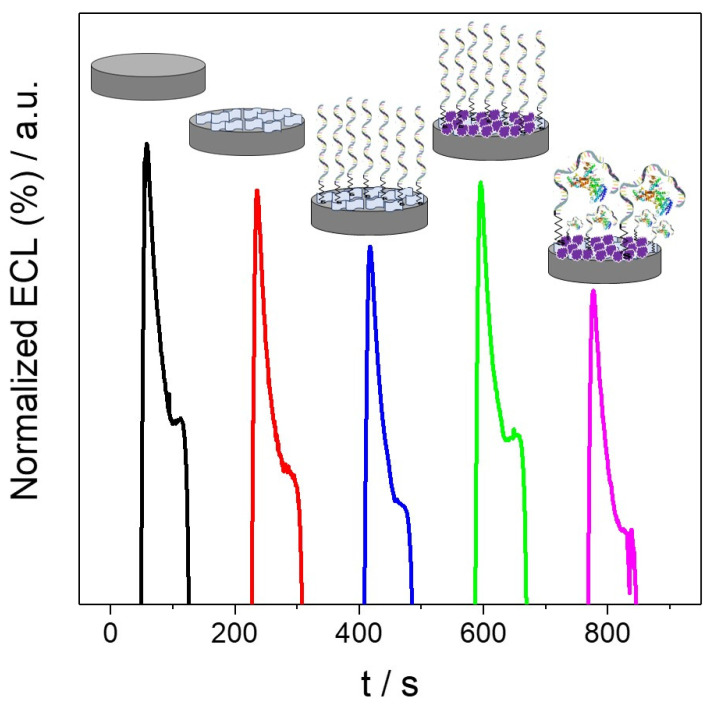
ECL signals of CSPE (black curve), CSPE/MoS_2_-NS (red curve), CSPE/MoS_2_-NS/Apt-SH (blue curve), CSPE/MoS_2_-NS/Apt-SH/BSA (green curve) and CSPE/MoS_2_-NS/Apt-SH/BSA/HER2 (purple curve; HER2 tested concentration was 13.7 pg/mL) using a solution of 7 mM [Ru(bpy)_3_]^2+^ and 8 mg/mL CDs in 0.2 M PB pH 8. Cyclic potential scan from 0.50 V to 1.40 V and 10 mV/s of scan rate.

Once the biosensor is characterized, we proceeded to study the ECL behavior of the aptasensor at each modified stage. The resulting ECL signals were normalized with the blank (CSPE/MoS_2_-NS/Apt-SH/BSA) and represented as a function of time (Figure 6). As can be seen, the ECL signal decreases after the nanostructuration (red curve) and aptamer immobilization (blue curve) stages whereas there is an increment in the ECL response when the platform is blocked with BSA (green curve). Finally, the incubation of the platform with the HER2 protein produces a decrease in the aptasensor response. This can be explained by the formation of the folded conformation of the aptamer after the recognition with HER2 protein, which generates a steric hindrance that impedes the access of the [Ru(bpy)_3_]^2+^/CD system to the surface of the electrode and, as a result, the electron-transfer reaction. It is worth to note that no decrease in the signal is observed when there is not HER2 protein in the incubation solution. Therefore, these results demonstrate that the [Ru(bpy)_3_]^2+^/CD system used in the ECL technique can detect the highly specific protein–aptamer interaction.

Based on the results obtained, we proceeded to study the response of the aptasensor as a function of the concentration of the HER2 protein analyte. For this purpose, nanostructured carbon screen-printed electrodes modified with the thiolated aptamer and blocked with BSA (CSPE/MoS_2_-NS/Apt-SH/BSA) were incubated with solutions of different HER2 concentrations (see experimental section) and their ECL signals were recorded. Figure S5 shows the normalized ECL signals (with the blank CSPE/MoS_2_-NS/Apt-SH/BSA) obtained at each different HER2 concentration. As can be seen, there is a linear decrease in the normalized ECL response of the aptasensor versus the logarithm of the HER2 protein concentration. From this linear response of the aptasensor, a calibration curve was obtained (Figure S5) that fits the linear equation: Normalized ECL (%) = (6.3 ± 0.8) – (4.0 ± 0.2)·log[HER2] (R^2^ = 0.9926). The detection limit and the determination limit were calculated as the concentration that gave a signal equal to blank (CSPE/MoS_2_-NS/Apt-SH/BSA) plus three and 10 times the standard deviation of blank, respectively. Values of 1.84 and 6.07 fg/mL were obtained for the LOD and LOQ, respectively.

The reproducibility was estimated by quantifying 428 fg/mL HER2 protein with three different aptasensors prepared in the same manner. The calculated relative standard deviation (RSD) was 1.3%.

The selectivity of the ECL aptasensor was also assessed. For this purpose, other proteins (CEA and p53) and a non-complementary DNA sequence (see Table 1) were tested as interferences. The normalized ECL response obtained by using each interference were compared with the signal obtained only with HER2 at a final concentration of 1.71 pg/mL (see Figure S6). As can be seen, non-complementary DNA sequence does not have interference on the detection of HER2, indicating the good selectivity of the aptasensor even in the presence of this potential interferent. However, in the case of P53 protein, a little interference is observed in the experimental conditions; in the case of CEA protein, a higher interference is observed. It is worth to note that in the case of the potential interferent proteins the concentration of both proteins is higher (100 ng/mL and 1.0 ng/mL for CEA and p53, respectively) than those normally found in plasma samples [61].

We have also evaluated the applicability of the aptasensor using a more complex matrix such as human serum sample. Hence, we evaluate the ECL signal response of three different aptasensors (n = 3), prepared in the same manner, using a spiked human serum sample (final HER2 concentration of 1.71 pg/mL) as analyte. After Including this ECL signal in the calibration plot, the HER2 concentration was 1.69 pg/mL and consequently the recovery was found to be 98.8%.

Finally, the storage stability of the CSPE/MoS_2_-NS/Apt-SH/BSA was also evaluated. The aptasensor (stored at 4 °C) kept a 93% of the initial ECL response for 45 days.

The developed aptasensor has been also compared with another ECL or electrochemical aptasensors for HER2 detection. As can be observed in Table 2, the ECL biosensor developed exhibits a lower detection limit than those described in the literature. It also has a wide linear concentration range which compares well with those previously reported in the literature. Furthermore, the method is simple, without the need for complex approaches or labeling steps. So, in conclusion, the developed aptasensor is a simple and rapid tool for HER2 detection proving a great alternative to the classical methods for diagnosing HER2 overexpressing breast cancer.

## 4. Conclusions

An ECL aptasensor for HER2 detection was successfully developed by using of MoS_2_-NS as immobilization platform of the biorecognition element and CDs as coreactants of [Ru(bpy)_3_]^2+^ anodic ECL. [Ru(bpy)_3_]^2+^/CD system was used for the first time to detect the highly specific protein–aptamer interaction using CSPEs nanostructured with MoS_2_-NS. Based on the present strategy, HER2 protein could be detected in a wide linear range (from 6.08 fg/mL to 13.7 pg/mL) directly by the change in ECL signal with a low detection limit of 1.84 fg/mL. Considering that breast cancer patients have elevated HER2 blood concentrations (15–75 ng/mL) in comparison with those observed for normal individuals (2–15 ng/mL) [62], this methodology allows discrimination between a healthy patient and a patient with HER2 overexpression and, therefore, at risk of breast cancer. In addition, this method represents a new, simple and versatile approach for biomarker proteins detection related to breast cancer disease.

## Figures and Tables

**Table 1:** DNA sequences used in the present work.

Nomenclature	Oligonucleotide Sequence
Apt-SH	5′-SH-(CH_2_)_6_-AACCGCCCAAATCCCTAAGAGTCTGCACTTGTCA TTTTGTATATGTATTTGGTTTTTGGCTCTCACAGACACACTACACACGCACA-3′
Apt-TAMRA-SH	5′-SH-(CH_2_)_6_-AACCGCCCAAATCCCTAAGAGTCTGCACTTGTCA TTTTGTATATGTATTTGGTTTTTGGCTCTCACAGACACACTACACACGCACA-TAMRA-3′
Interference DNA sequence	AGTGAGTGCGGTTAGACCTGCTAGG

**Table 2:** Electrochemiluminescence (ECL) and electrochemical aptasensors for human epidermal growth factor receptor 2 (HER2) detection.

Principle	Method	Target Analyte	Linear Range	L.O.D.	Reference
Reduced graphene oxide nanosheets and rhodium nanoparticles	DPV	Extracellular domain of HER2	10.0 to 500.0 ng/mL	0.667 ng/mL	[14]
Au nanoparticles	DPV	HER2	0.001 to 100 ng/mL	0.001 ng/mL	[15]
Exonuclease recycling amplification and host–guest recognition	DPV	HER2	10 ng/mL to 150 ng/mL	4.9 ng/ml	[16]
Graphene quantum dots, polypyrrole and cobalt phthalocyanine	EIS	HER2	1 to 10 ng/mL	0.00141 ng/mL	[17]
Bimetallic ZrHf-based metal-organic framework embedded with carbon dots	EIS	HER2	0.001 to 10 ng/mL	19 fg/mL	[18]
A novel binary luminophore (Zr-MOF modified bulk boron carbon oxynitride)	ECL	HER2	10 fg/mL to 100 ng/mL	0.2 fg/mL	[19]
CDs and MoS_2_-NS	ECL	HER2	6.08 fg/mL to 13.7 pg/mL	1.84 fg/mL	Present work

## Data Availability

The data presented in this study are available on request from the corresponding author.

## Supplementary Materials

The following supporting information can be downloaded at: https://www.mdpi.com/article/10.3390/bios13030348/s1. Figure S1. (A) Transmission Electron Microscopy (TEM) images, (B) size histogram, (C) infrared absorption spectrum and (D) elemental analysis of the obtained carbon nanodots (CDs). Figure S2. (A and B) Scanning Electron Microscopy (SEM) images of MoS_2_-NS taken with a model VERIOS 460 from FEI. (C) Extinction and (D) Raman spectra (laser excitation 532 nm) of MoS_2_-NS (the insert corresponds to a broadening of the spectra in the region of 350 to 438 cm^−1^). Figure S3. Fluorescence spectrum of 20 µM [Ru(bpy)_3_]^2+^ in 0.2M PB pH 8 obtained exciting at the maximum absorption wavelength of [Ru(bpy)_3_]^2+^ specie (λ_exc_ = 286 nm). Figure S4. (A) Secondary and (B) backscattered electrons SEM images of bare Carbon Screen Printed Electrode (CSPE). Figure S5. Calibration curve (n = 3) of the normalized ECL response of the aptasensor vs. log HER2 concentration (from 428 fg/mL to 13.7 pg/mL). Same experimental conditions than Figure 6. Figure S6. Interference study (n = 3, three different platforms): ECL aptasensor response in presence of a solution of 1.71 pg/mL HER2 (black bar), 1.71 pg/mL HER2 and 1 µM non-complementary DNA sequence (grey bar), 1.71 pg/mL HER2 and100 ng/mL CEA (blue bar) and 1.71 pg/mL HER2 and 1.0 ng/mL p53 (red bar).
