# Supplementary material for: MoS2-Carbon Nanodots as a New Electrochemiluminescence Platform for Breast Cancer Biomarker Detection
Source: Biosensors (Basel). 2023 Mar 5;13(3):348. doi: 10.3390/bios13030348 (PMC10046281; doi:10.3390/bios13030348)
Supplement: Supplementary file 1 [file biosensors-13-00348-s001.zip › biosensors-2225244-supplementary.pdf]

biosensors

*Supplementary Material*

# MoS2-Carbon Nanodots as New Electrochemiluminescence Platform for Breast Cancer Biomarker Detection

**Laura Gutiérrez-Gálvez [1], Manuel Vázquez Sulleiro [2], Cristina Gutiérrez-Sánchez [1], Daniel García-Nieto [3], Mónica Luna [3], Emilio M. Pérez [2], Tania García-Mendiola [1,4,*] and Encarnación Lorenzo [1,2,4]**

[1] Departamento de Química Analítica y Análisis Instrumental. Universidad Autónoma de Madrid. 28049, Madrid, Spain
[2] IMDEA-Nanociencia, Ciudad Universitaria de Cantoblanco. 28049, Madrid, Spain
[3] Instituto de Micro y Nanotecnología IMN-CNM, CSIC (CEI UAM+CSIC), Isaac Newton 8. Tres Cantos. 28760, Madrid, Spain
[4] Institute for Advanced Research in Chemical Sciences (IAdChem). Universidad Autónoma de Madrid. 28049, Madrid, Spain
* Correspondence: tania.garcia@uam.es

**Citation:** Gutiérrez-Gálvez, L.; Sulleiro, M.V.; Gutiérrez-Sánchez, C.; García-Nieto, D.; Luna, M.; Pérez, E.M.; García-Mendiola, T.; Lorenzo, E. MoS2-Carbon Nanodots as New Electrochemiluminescence Platform for Breast Cancer Biomarker Detection. *Biosensors* **2023**, *13*, 348. https://doi.org/10.3390/bios13030348

Received: 1 February 2023
Revised: 23 February 2023
Accepted: 2 March 2023
Published: 5 March 2023

**Copyright:** © 2023 by the authors. Licensee MDPI, Basel, Switzerland. This article is an open access article distributed under the terms and conditions of the Creative Commons Attribution (CC BY) license (https://creativecommons.org/licenses/by/4.0/).

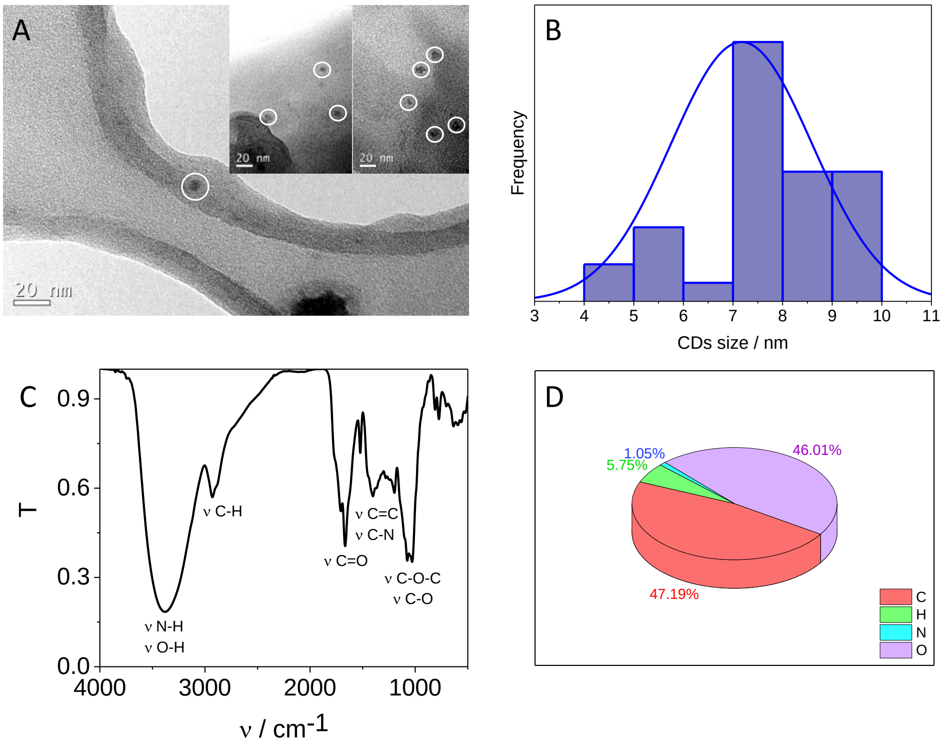


**Figure S1.** (A) Transmission Electron Microscopy (TEM ) images, (B) size histogram, (C) infrared absorption spectrum and (D) elemental analysis of the obtained carbon nanodots (CDs).

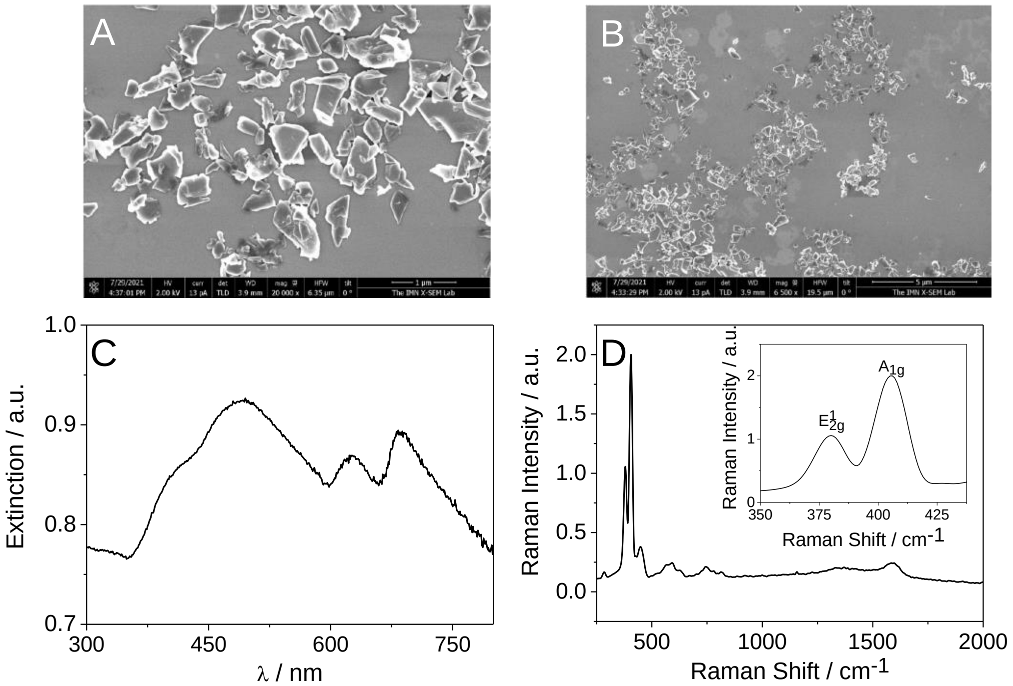


**Figure S2.** (A and B) Scanning Electron Microscopy (SEM) images of $MoS_2$-NS taken with a model VERIOS 460 from FEI. (C) Extinction and (D) Raman spectra (laser excitation 532 nm) of $MoS_2$-NS (the insert corresponds to a broadening of the spectra in the region of 350 to 438 $cm^{-1}$).

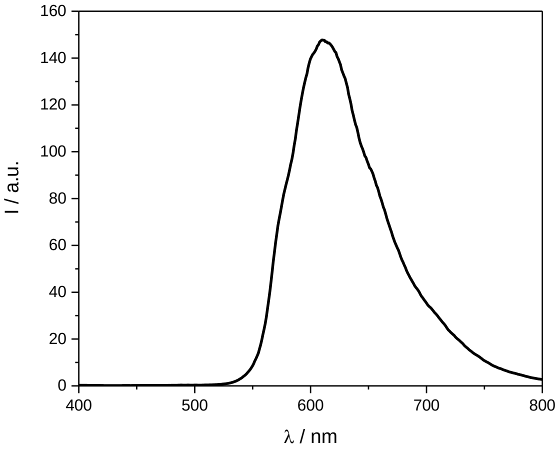


**Figure S3**. Fluorescence spectrum of 20 μM $[Ru(bpy)_3]^{2+}$ in 0.2M PB pH 8 obtained exciting at the maximum absorption wavelength of $[Ru(bpy)_3]^{2+}$ specie ($\lambda_{exc}$ = 286 nm).

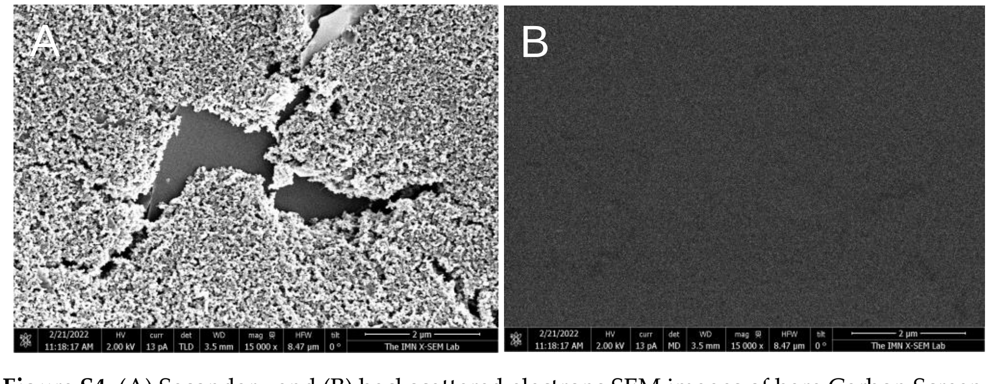


**Figure S4.** (A) Secondary and (B) backscattered electrons SEM images of bare Carbon Screen Printed Electrode (CSPE).

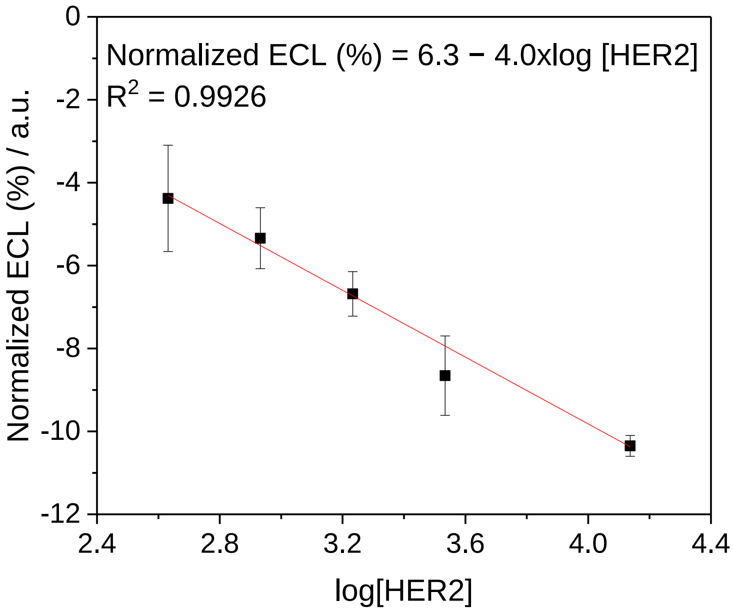


**Figure S5**. Calibration curve (n=3) of the normalized ECL response of the aptasensor vs log HER2 concentration (from 428 fg/mL to 13.7 pg/mL). Same experimental conditions than Figure 6.

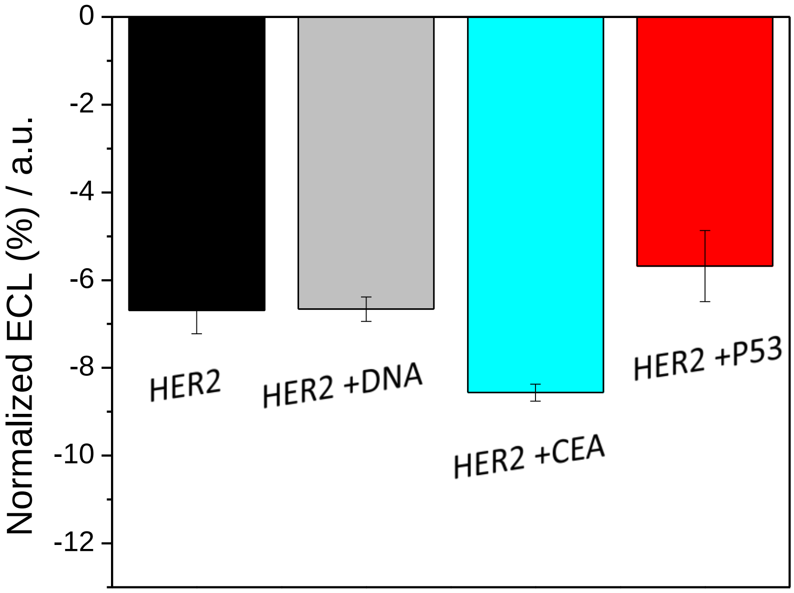


**Figure S6.** Interference study (n=3, three different platforms): ECL aptasensor response in presence of a solution of 1.71 pg/mL HER2 (black bar), 1.71 pg/mL HER2 and 1 μM non-complementary DNA sequence (grey bar), 1.71 pg/mL HER2 and100 ng/mL CEA (blue bar) and 1.71 pg/mL HER2 and 1.0 ng/mL p53 (red bar).
